# Widespread insecticide resistance in *Aedes aegypti L*. from New Mexico, U.S.A.

**DOI:** 10.1371/journal.pone.0212693

**Published:** 2019-02-22

**Authors:** Yashoda Kandel, Julia Vulcan, Stacy D. Rodriguez, Emily Moore, Hae-Na Chung, Soumi Mitra, Joel J. Cordova, Kalli J. L. Martinez, Alex S. Moon, Aditi Kulkarni, Paul Ettestad, Sandra Melman, Jiannong Xu, Michaela Buenemann, Kathryn A. Hanley, Immo A. Hansen

**Affiliations:** 1 Department of Biology, New Mexico State University, Las Cruces, NM, United States of America; 2 New Mexico Department of Health, Santa Fe, NM, United States of America; 3 Department of Geography, New Mexico State University, Las Cruces, NM, United States of America; Texas A&M University College Station, UNITED STATES

## Abstract

**Background:**

*Aedes aegypti* mosquitoes are vectors of a variety of emerging viral pathogens, including yellow fever, dengue, chikungunya, and Zika virus. This species has established endemic populations in all cities across southern New Mexico sampled to date. Presently, control of *Aedes*-borne viruses relies on deployment of insecticides to suppress mosquito populations, but the evolution of insecticide resistance threatens the success of vector control programs. While insecticide resistance is quite common in *Ae*. *aegypti* field populations across much of the U.S., the resistance status of this species in populations from New Mexico has not previously been assessed.

**Results:**

First, we collected information on pesticide use in cities in southern New Mexico and found that the most commonly used active ingredients were pyrethroids. The use of insecticides with the same mode-of-action over multiple years is likely to promote the evolution of resistance. To determine if there was evidence of resistance in some cities in southern New Mexico, we collected *Ae*. *aegypti* from the same cities and established laboratory strains to assess resistance to pyrethroid insecticides and, for a subset of populations, to organophosphate insecticides. F2 or F4 generation mosquitoes were assessed for insecticide resistance using bottle test bioassays. The majority of the populations from New Mexico that we analyzed were resistant to the pyrethroids permethrin and deltamethrin. A notable exception to this trend were mosquitoes from Alamogordo, a city that did not report using pyrethroid insecticides for vector control. We screened individuals from each population for known knock down resistance (kdr) mutations via PCR and found a strong association between the presences of the F1534C kdr mutation in the *para* gene of *Ae*. *aegypti* (homologue to F1534C in *Musca domestica L*.) and pyrethroid resistance.

**Conclusion:**

High-level pyrethroid resistance is common in *Ae*. *aegypti* from New Mexico and geographic variation in such resistance is likely associated with variation in usage of pyrethroids for vector control. Resistance monitoring and management is recommended in light of the potential for arbovirus outbreaks in this state. Also, alternative approaches to mosquito control that do not involve insecticides should be explored.

## Background

*Aedes aegypti* is an anthropophilic mosquito species with a worldwide distribution in tropical, subtropical and temperate climate zones [1]. It is the principal vector of yellow fever, dengue fever, chikungunya, and Zika virus [2, 3]. This species originated in Africa and has been transported around the world by humans first in sailing ships and later in trains, automobiles, and airplanes [4]. *Ae*. *aegypti* has infested all southern states of the U.S. [5]. In New Mexico, *Ae*. *aegypti* is common in cities in the southern counties of the state but is absent in the central portion of the state north of Albuquerque [1]; the distribution of this species in northeastern New Mexico is still under investigation. The populations of *Ae*. *aegypti* in New Mexico are closely related to other populations of the species in southwestern North America [6]. As is typical for *Ae*. *aegypti* in southwestern deserts, [7–10], *Ae*. *aegypti* populations in New Mexico undergo drastic seasonal fluctuations driven by the annual monsoon (authors’ personal observations). While no local transmission of *Ae*. *aegypti*-borne diseases have been reported in New Mexico, the high densities of this mosquito in urban areas coupled with frequent introductions of these viruses into the U.S. are a cause for concern [11–14].

Insecticides are the most common and the most successful tool used to control human disease vectors [15]. They are widely used in New Mexico [16]. However, insecticide resistance is a serious problem for mosquito control efforts [17]. Insecticide resistance evolves readily in insect populations when they are repeatedly exposed to the same insecticide or to insecticides with the same mode of action. Such resistance undermines effort to improve public health. For instance, high levels of resistance severely impede malaria control efforts in Africa [18, 19] and interventions to control dengue and Zika virus transmission in South America [20, 21].

The mechanisms of mosquito insecticide resistance fall into two broad categories: enhanced metabolic detoxification of insecticides [22] and mutations in insecticide target proteins that render these proteins less susceptible to corresponding insecticides [23, 24]. As examples of the latter mechanism, knock down resistance (kdr) mutations in the *paralytic (para)* gene of the house fly *Musca domestica* and the fruit fly *Drosophila melanogaster* can render these two dipteran species highly resistant to pyrethroid insecticides [25–27]. The *para* gene encodes a voltage-gated sodium channel that is expressed in axons of nerve cells and that is the main target of pyrethroids. Pyrethroid-binding prevents these channels from closing, resulting in the complete depolarization of the axonal membrane, paralyzing the insect. Specific point mutations in the *para* gene that alter sodium channel structure in such a way that there is decreased affinity towards pyrethroids can cause insecticide resistance in mosquitoes by reducing neuronal sensitivity to this class of insecticides. The *dipteran* PARA protein is a large channel with 24 transmembrane domains organized in four domains (I-IV) each with six membrane spanning segments [28]. Several point mutations in the para gene of *Aedes aegypti* have been implicated in pyrethroid resistance. In particular the mutations V410L, G923V, L982W, S989P, I1011M, I1011V, V1016G, V1016I, T1520I, F1534C, and D1763Y are known to confer the kdr phenotype [28–34]. The various mutations in the Para protein are numbered differently in different insect species due to variation in the length of the homologous proteins. For example, *Ae*. *aegypti* F1565 is the homologous mutation to *Musca domestica* F1534C and *Aedes albopictus* F1474C [35]. We use the *Musca* annotation numbers in this manuscript since this is established practice.

*Aedes aegypti* KDR mutations with high allele frequency have been found in several countries, including the US. For example, the F1534C KDR mutation was reported to occur at a frequency of 0.41–0.79 in India [36], while F1534C and a second KDR mutation, V1016G, were detected in insecticide resistant *Aedes aegypti* across Malaysia [37]. The F1534C mutation was detected at high frequency in *Ae*. *aegypti* from Brazil and Thailand as well [33, 38]. A DDT and pyrethroid-resistant population of *Ae*. *aegypti* from the Grand Cayman islands carried the F1534C mutation at an allele frequency of 0.68 [39]. F1534C is also common in *Ae*. *aegypti* from Mexico [40, 41]. Taken together, these studies suggest that the F1534C mutation is one of the most common and widespread kdr mutations in *Ae*. *aegypti*.

In the current study, we characterized the pattern of pyrethroid resistance in seven populations of *Aedes aegypti* established from mosquitoes collected in cities in southern New Mexico and screened for resistance mutations in the para gene, the molecular target of pyrethroids. We found that resistance was widespread in the offspring of the mosquitoes we collected and associated with a high frequency of the kdr mutation F1534C. Importantly, Alamogordo, which was also the only city that did not report use of pyrethroids for mosquito control, was also the only city in which *Ae*. *aegypti* did not exhibit strong resistance to pyrethroids.

## Materials & methods

### Collection of pesticide usage data

In New Mexico, the entity responsible for vector control varies by county and city. Municipal offices in each city listed in **Table A in S1 File** or its home county were contacted via email and/or telephone to request the name and contact information of the individual or office that could provide information on pesticides used for mosquito control. We then requested that those individuals provide us with a list of pesticides in use in 2017 as well as those used over the previous five years, if possible. We were unable to obtain contacts for Roswell or Portales, and Lordsburg does not provide vector control. In many cases, vector control professionals were able to provide specific ingredients (active chemicals) in use, but not pesticide product name *per se*. Of the cities queried, only Alamogordo reported that they did not use chemical pesticides; they utilize the bacterial larvicide Vectobac. We note that this inquiry did not capture vector control contractors or other similar entities.

### Establishment of New Mexico *Ae*. *aegypti* strains

Mosquito adults and eggs were collected in the summer and early fall of 2017 as part of the SouthWest *Aedes* Research and Mapping project (SWARM). Adults were collected using BG Sentinel or gravid traps. Adults were transferred into BugDorm-1 Insect Rearing Cage (30 by 30 by 30 cm, BugDorm Store, Taichung, Taiwan) and given 20% sucrose ad libitum. The number of founding individuals and the number of different locations where trapping was performed is shown in **Table B in S1 File**. Despite vigorous collection efforts, it was not possible to collect a sufficient number of field-derived mosquitoes to immideately test insecticide resistance. Thus propagation of these strains was performed as describe below in the mosquito culture section. Eggs were collected using dark oviposition cups containing water and a wooden spatula [42]. Mosquito eggs were collected, dried, and stored for at least three days before hatching the G0 generation. Strains from different cities were amplified to F2 or, in some cases, F4 generation to produce enough individuals to perform Resistance Bottle Tests (see below). We were careful to keep the number of generations well below the 10 generations required to restore pyrethroid susceptibility in Ae. *aegypti* [43]. We were able to collect enough adult mosquitoes from eight cities in Southern New Mexico: Alamogordo, Carlsbad, Deming, Las Cruces, Lovington, Roswell, and Sunland Park to establish laboratory strains with sufficient numbers to perform insecticide resistance testing at the F2 (N = 4 cities) or F4 (N = 3 cities). We were not successful in establishing a laboratory strain with mosquitoes collected in Socorro, but were able to genotype these field-collected mosquitoes (below).

### Mosquito culture

Mosquito culture protocols were similar to those described in Marquardt, 2004 [44]. Larvae were reared in large pans containing 1.5L of distilled water. The larvae were fed ad libitum with Special Kitty cat food (Amazon). Each pan received approximately 4 pellets and the water was changed every 3 days. This regimen ensured that larvae were not crowded, as high larval densities can suppress insecticide resistance [45]. When pupae started to appear, they were removed daily and placed into small cups with water within BugDorm-1 Insect Rearing Cages (Bugdorm Store, Mega View Science, Taiwan). Cages with the adults were maintained in an insectary at 80% humidity and 27°C with a light/dark cycle of 14/10, respectively. Adult female mosquitoes were blood-fed via an artificial feeding system (Chemglass Life Sciences, Vineland, NJ) with bovine blood purchased from a commercial provider (Hemostat, Dixon, CA). Mosquitoes were offered a blood meal twice a week to ensure high egg yields. Three days after blood feeding a cup with water lined with partially submerged germination paper was put in the cage to serve as an egg laying substrate. Eggs were collected after three days, dried and stored in paper envelopes in an insect chamber at 27°C and 80% humidity.

### Insecticide resistance bottle tests

Insecticide resistance was tested using a novel bottle bioassay that we developed based on the standard CDC Bottle Bioassay [46, 47] with major modifications. The protocol for this test was deposited at **https://www.protocols.io/** under the title ‘NMSU Mosquito Insecticide Resistance Bottle Test Protocol’. Insecticides were purchased from Sigma-Aldrich (see Table 1). Bottles were prepared 24 hours prior to the experiment tests with one of four insecticides chosen based on the pesticide usage data shown in Table 1: the pyrethroids etofenprox (37.5μg/bottle), permethrin (86μg/bottle), deltamethrin (10.5μg/bottle) and the organophosphate chloropyrifos (40μg/bottle). These concentrations, chosen based on a pilot calibration experiment for each active ingredient, are higher than recommended in the original protocol, and therefore reflect a highly conservative test of resistance. Moreover, these higher concentrations allow effective detection of insecticide resistance in a shorter period than the standard bottle test. Bottles rinsed in acetone were used as a control. Two control mosquito strains were procured from MR4 [48]: the Rockefeller laboratory strain (Rock, MRA-734), which is sensitive to pyrethroids and was used as a negative control and the Puerto Rico strain (Puerto Rico, NR-48830), which is resistant to pyrethroids and was used as a positive control. A 50:50 mixture of individuals of ROCK and Puerto Rico mosquitoes was also used as an additional control to represent populations which contained a mixture of resistant and susceptible mosquitoes. Fifteen unfed mosquitoes at 2–5 days old were added to each bottle. Mortality over time was assessed by visual observation. Mosquitoes that were unable to crawl were counted as dead. Nine replicates were performed for each treatment. Statistical differences between the mortality curves of the control strains and the experimental strains were assayed via Kaplan Meier survival analysis [49] using XLSTAT. Strains that showed significantly delayed mortality (p< = 0.05) were identified as resistant.

**Table 1:** Pesticides used in this study.

Name	Sigma catalog #	CAS-No.	Concentration (%)
Etofenprox	34094	80844-07-1	< = 100
Permethrin	45614	52645-53-1	90–100
Deltamethrin	45423	52918-63-5	90–100
Chlorpyrifos	45395	2921-88-2	< = 100

### Genotyping kdr mutations in the *para* gene

DNA from individual mosquitoes was extracted using the DNeasy Blood & Tissue Kit following the manufacturer’s instructions (Qiagen, Palo Alto, CA). Homogenization was performed with disposable pellet mixers and a cordless motor (VWR, Radnor, PA). PCRs primers for the sequences surrounding amino acid positions 989, 1534, and 1763 of the *Ae*. *aegypti* voltage-gated para-like sodium channel *(para)* (Genbank accession number >EU399181.1) were designed using Primer Blast software [50] on the website of the National Center for Biotechnology Information (NCBI); primer design was further refined based on primers used in previous studies [35, 51, 52].

Primers used in this study are shown in **Table 2**. The PCR amplicon from the first primer pair includes three relevant mutation sites: S989P, I1011M, and L1021F/H/S while the amplicons derived from the other two primer pairs cover one mutation site each: F1534C, and D1763Y. We used the Taq PCR Master Mix Kit (Qiagen, Hilden, Germany). PCR reactions contained 10 to 100 ng of genomic DNA. PCR conditions were identical for all three amplicons: 2 minutes at 95°C, followed by 35 cycles of 30 seconds at 95°C, 30 seconds at 60° C, and 30 seconds at 68° C, followed by the final extension for 10 minutes at 68° C and the final holding temperature of 10° C. PCR products were cleaned using the QIAquick PCR purification kit (Qiagen). DNA fragments were sent to MCLAB (Molecular Cloning Laboratories, South San Francisco, CA) for Sanger sequencing using the forward primers in the table. DNA sequencing files were analyzed using Seqman software (DNAstar, Mdison, WI).

**Table 2:** Primers used in this study.

Primer Name	Sequence
989f	5’ GAC AAT GTG GAT CGC TTC CC 3’
989r	5’ TGC CGA CAG CGA GGA TGA ACC 3’
1534f	5’ GAG AAC TCG CCG ATG AAC TT 3’
1534r	5’ TCT GCT CGT TGA AGT TGT CGA T 3’
1763f	5’ TCG AGA AGT ACT TCG TGT CG 3’
1763r	5’ AAC AGC AGG ATC ATG CTC TG 3’

## Results

### Pesticide use in New Mexico

We requested information from personnel that were responsible for vector control from twelve cities in New Mexico and received a total of ten answers (**Table A in S1 File**). Pyrethroids are the most common public health insecticides currently in use in New Mexico, and chlorpyrifos is also a commonly used active ingredient. Only Alamagordo reported that they did not use adulticides, relying instead on VectoBac to target larvae.

### Resistance of mosquitoes from New Mexico to permethrin and other insecticides

**Fig 1:**
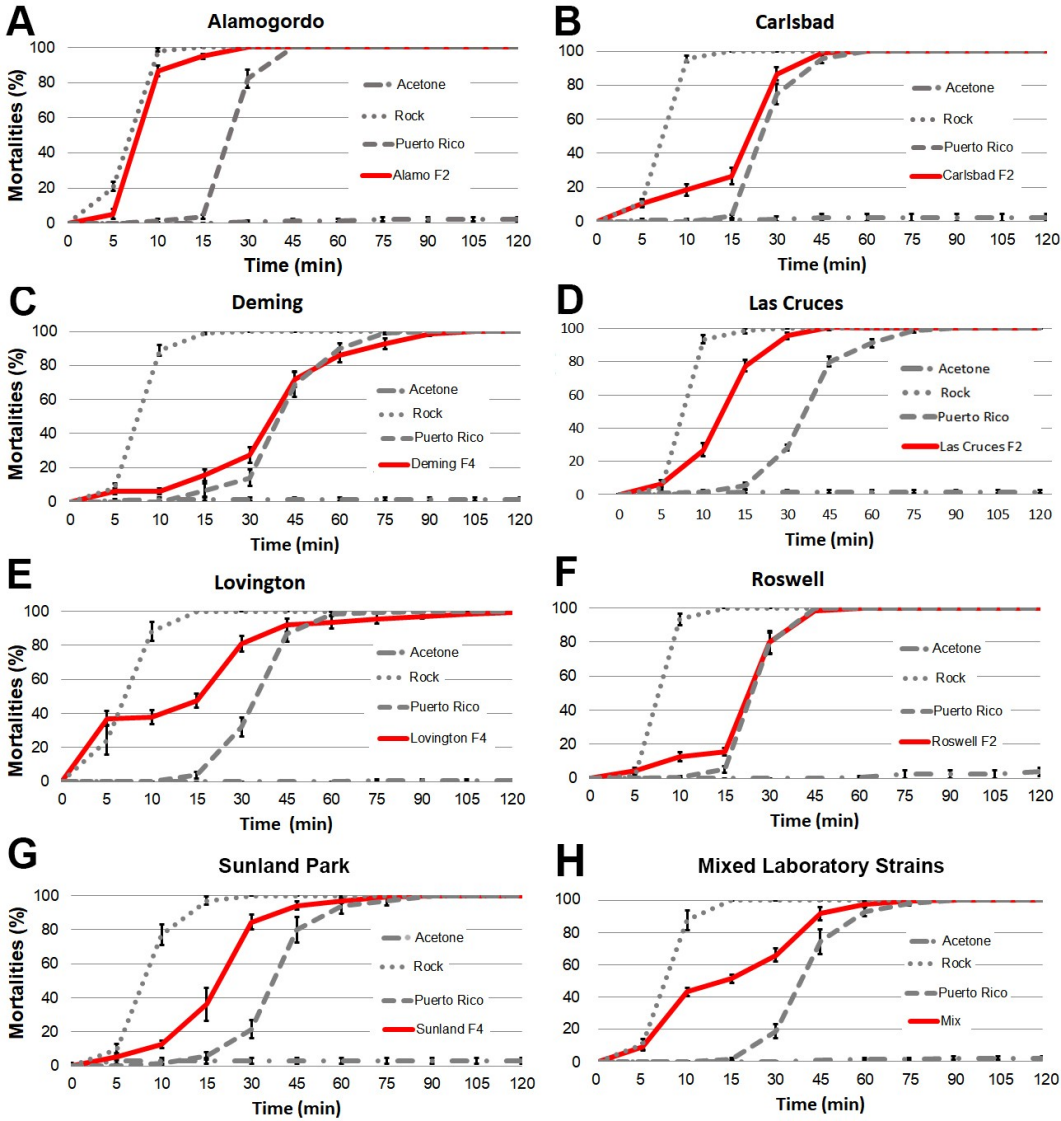
Permethrin-resistance levels in *Aedes aegypti* from New Mexico. Shown are mortality curves of a permethrin-susceptible control strain (Rock, dotted line), a resistant control strain (Puerto Rico, dashed line), susceptible mosquitoes in acetone-treated bottles (Rock, dotted/dashed line), and strains established from field collections (red line). Please note that the X-axis is not a linear time scale but has five minute intervals until 15 min and then switches to 15 min intervals. In all cases **(A-H)**, the control (Acetone) mortality curves were significantly different (P<0.0001) from the curves for all other treatments and the curves plotted for the Puerto Rico strain and the Rock strain were also significantly different from each other (P < .0001). The curves plotted for the permethrin-exposed Rock strain were also significantly different from the other strains (P < .0001). **A.** The curve plotted for the Puerto Rico strain (a known resistant strain) is significantly different than that plotted for the Alamogordo strain (P < .0001). **B.** The curve plotted for the Puerto Rico strain is significantly different than that plotted for the Carlsbad strain (P < .0001). **C.** The curve plotted for the Puerto Rico strain is not significantly different than that plotted for the Carlsbad strain (P = 0.597). **D.** The curve plotted for the Puerto Rico strain is significantly different than that plotted for the Las Cruces strain (P < .0001). **E.** The curve plotted for the Puerto Rico strain is not significantly different than that plotted for the Lovington strain (P < .0001). **F**. The curve plotted for the Puerto Rico strain is not significantly different than that plotted for the Roswell strain (P = .206). **G.** The curve plotted for the Puerto Rico strain is not significantly different than that plotted for the Sunland strain (P < .0001). **H.** There was a significant difference between the curves plotted for the permethrin-exposed mixed strain (50% Rock, 50% Puerto Rico) and the curves plotted for the pure strains (P < .0001). The curve plotted for the Puerto Rico strain is not significantly different than that plotted for the mixed strains (P < .0001).

**Fig 2:**
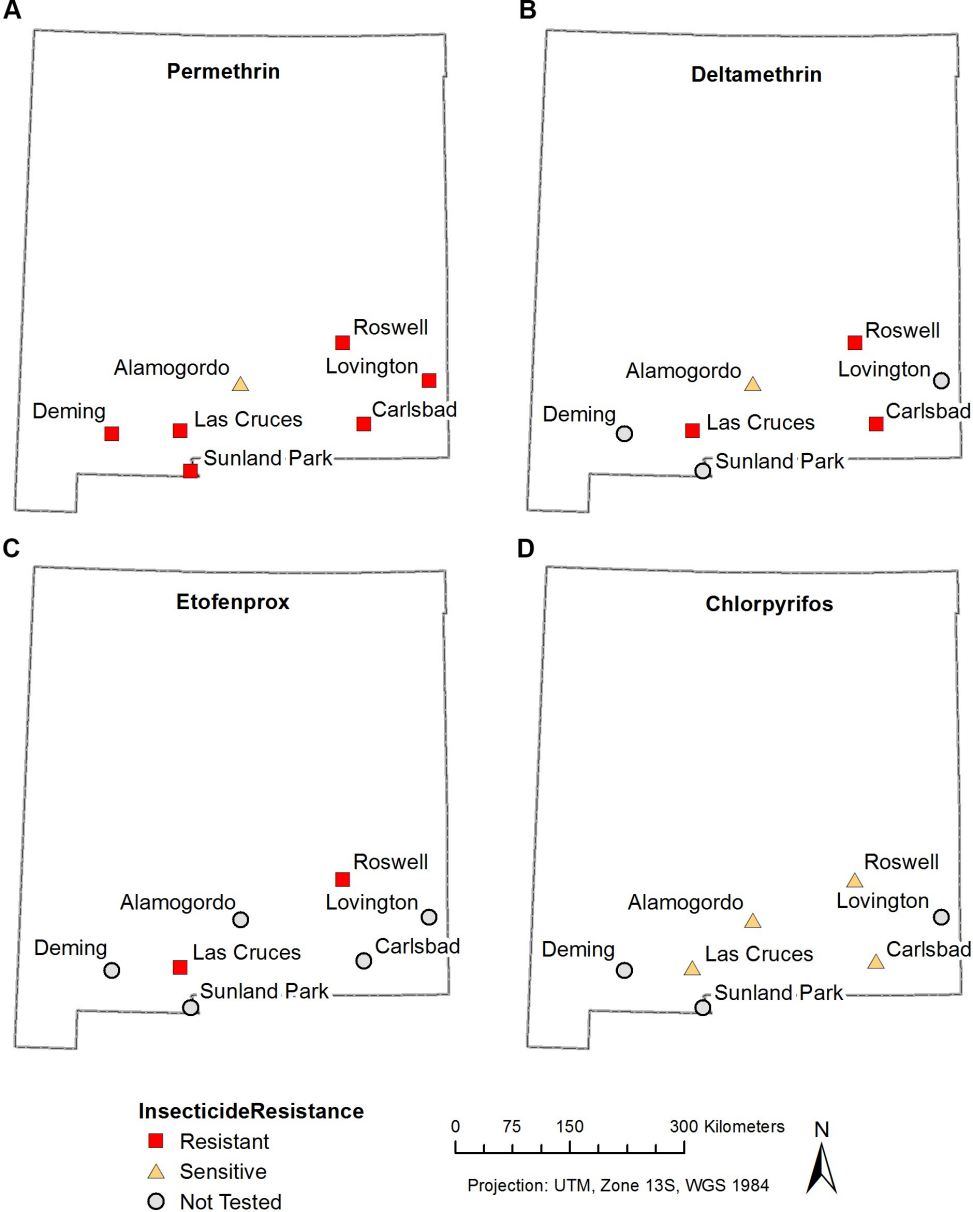
Insecticide resistance in *Aedes aegypti* from New Mexico. The resistance status of individual populations was determined via bottle testing (see **Fig 1** and **Figs A-J in S1 File**). Populations were classified as resistant when their mortality curves were significantly shifted towards longer survival compared to the mortality curves of the sensitive Rock strain. **Resistant** populations are marked with **red** squares, **sensitive** populations are marked with **yellow** triangles. The grey circles represent populations that have not yet been tested for this particular resistance. ALA–Alamogordo, CRL- Carlsbad, DEM-Deming, LC-Las Cruces, LOV-Lovington, ROS-Roswell, SOC-Socorro, SUN-Sunland Park. **A.** resistance map for permethrin, **B.** resistance map for deltamethrin, **C.** resistance map for etofenprox, **D.** resistance map for chlorpyrifos.

We performed a novel insecticide resistance bottle test to determine mortality curves with field-derived *Ae*. *aegypti* strains from New Mexico in order to determine whether they exhibited levels of resistance comparable to a highly resistant strain, the Puerto Rico strain, for permethrin **(Fig 1**), deltamethrin, and etofenprox **(Figs A–F in S1 File**). Subsequently we tested pyrethroid-resistant strains for resistance against chlorpyrifos (**Figs G—J in S1 File**). Due to limitations in the number of mosquitoes available, not every New Mexico strain was tested against every insecticide. The *Ae*. *aegypti* laboratory strains **Rockefeller** (Rock) and **Puerto Rico** showed, as expected, no resistance and strong resistance, respectively, to pyrethroids (**Fig 1, Figs A—F in S1 File**). **Fig 2** gives and overview of our findings, described below and in **Figs A–J in S1 File**, for each New Mexico strain. Susceptible Rockefeller mosquitoes showed high mortality rates after ten minutes while the Puerto Rico strain mosquitoes started to die after 30 minutes. There was variation when complete mortality was reached in the Puerto Rico strain ranging from 45 min to 75 minutes. Mosquitoes from **Alamogordo** (F2 generation) showed some resistance against permethrin and deltamethrin compared to the Rock strain. However, Alamogordo resistance levels were much lower than those found for the Puerto Rico control strain. Mosquitoes from Alamogordo showed no resistance against chlorpyrifos (**Fig 2, Fig A and H in S1 File)**. Mosquitoes from **Carlsbad** (F2 generation) showed high levels of resistance against permethrin and deltamethrin but were sensitive to chlorpyrifos (**Fig 1B**, **Figs C and I in S1 File).** Mosquitoes from **Deming** (F4 generation) showed high levels of resistance against permethrin (**Fig 1C**). Because of low mosquito numbers available, we did not perform tests for other insecticides. Mosquitoes from **Las Cruces** (F2 generation) showed resistance to permethrin, deltamethrin, and etofenprox while there was no resistance against chlorpyrifos. (**Fig 1D and Figs B, F, G in S1 File**). The levels of resistance against pyrethroids were intermediate to the levels measured for the two control strains (**Fig 1D)**. Mosquitoes from **Lovington** (F4 generation) were only tested against permethrin. Lovington mosquitoes’ survival curve was bimodal, suggesting that the mosquitoes we tested were a mixture of susceptible and highly resistant mosquitoes (**Fig 1E**). Supporting this interpretation, a mixture of mosquitoes from the laboratory strains **Rockefeller** (susceptible) and **Puerto Rico** (resistant) resulted in a double-sigmoid mortality curve (**Fig 1H**). Because of low mosquito numbers available, we did not perform tests for other insecticides. Mosquitoes from **Roswell** (F2 generation) showed strong resistance to permethrin, deltamethrin, and etofenprox while there was no resistance against chlorpyrifos (**Fig 1F, and Figs D, E, J in S1 File)**. Mosquitoes from **Sunland Park** (F4 generation) showed some resistance against permethrin (**Fig 1G**). Because of low mosquito numbers available, we did not perform tests for other insecticides.

### Kdr mutation frequencies in *Ae*. *aegypti* from New Mexico

**Fig 3:**
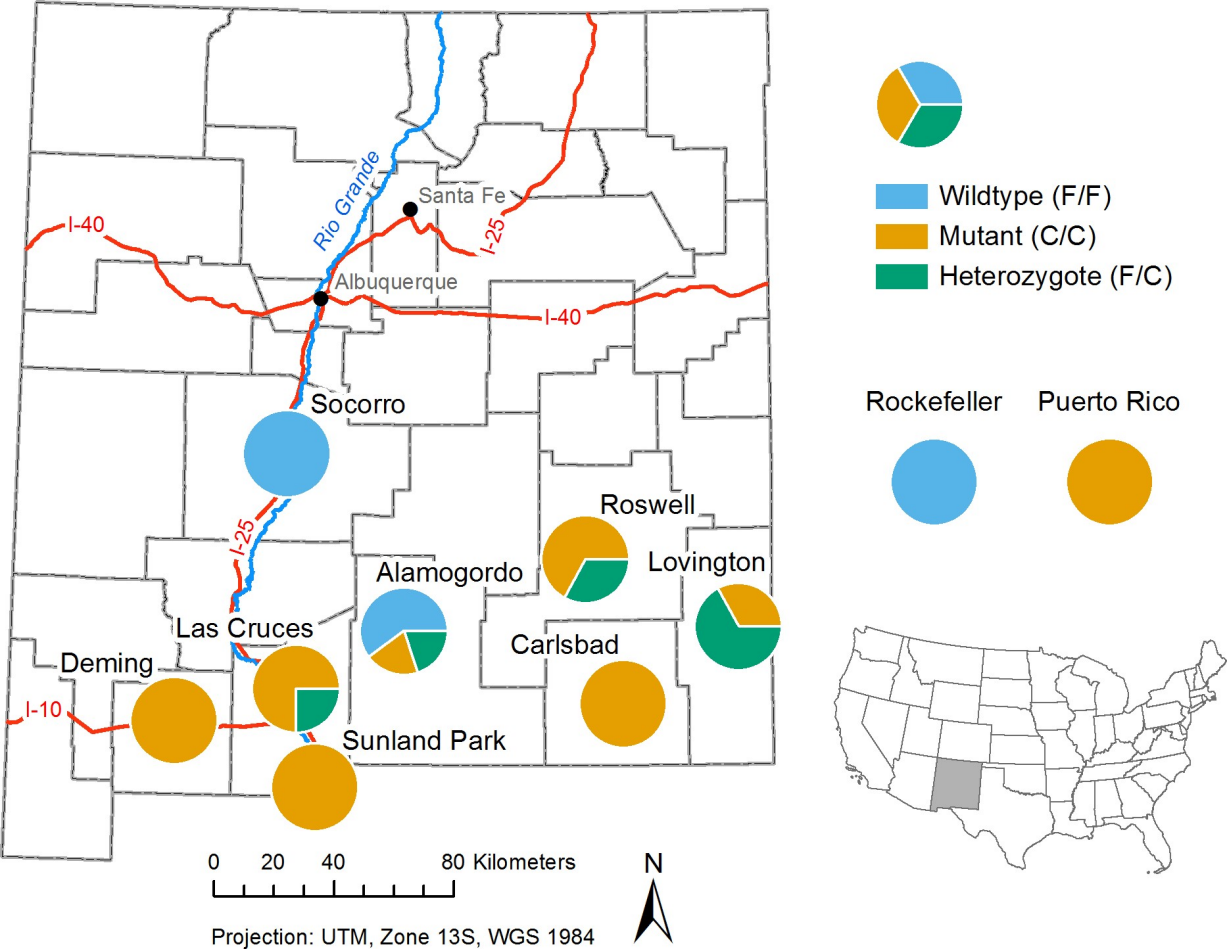
Genotype frequency map. The pie charts represent the percentage of the three specific kdr F1534C genotypes in mosquitoes collected at different locations. Wildtype, susceptible mosquitoes are shown in blue. Mutant, resistant mosquitoes are shown in yellow. Heterozygotes are shown in green. The genotype frequencies of the laboratory strains, Rockefeller and Puerto Rico, are shown to the upper right of the map. The location of New Mexico within the United States is shown in the lower right of the figure.

**Table 3** shows the genotypes of 50 individual mosquitoes from various locations in southern New Mexico. Mutations were observed only at the 1534 position of the kdr gene. Phenylalanine (F/F) at this position (wildtype) confers susceptibility to pyrethroids while a cysteine (C/C) confers resistance (mutant) in *Ae*. *aegypti* [28]. The resistance status of heterozygotes (F/C) is unknown. Out of 46 mosquitoes we analyzed, 29 (60%) were found to have the homozygous mutant phenotype (C/C), 8 (17%) were found to be heterozygous (F/C), and 11 (23%) were found to be wildtype (F/F) (**Fig K in S1 File**). **Fig L in S1 File** shows an alignment of the *Aedes aegypti* and *Musca domestica* Para proteins. **Fig 3** shows a genotype frequency map of the different genotypes we found for the 1534 mutation in different cities in New Mexico.

**Table 3:** kdr genotypes in mosquitoes from New Mexico–Shown are the genotypes of 50 individual mosquitoes collected at different locations in New Mexico during the 2017 mosquito season. The letters represent the specific amino acids encoded by the sequenced DNA at the specific locations (location numbers refer to the *Musca* para protein). Identical letters indicate that mosquitoes were homozygous at this position while different letters show that mosquitoes were heterozygous at this position. Genotypes of the pyrethroid-susceptible control strain Rockefeller and the pyrethroid-resistant Puerto Rico control strain are shown at the end of the table (the genotypes of five individual mosquitoes were identical in these strains). Amino acids are identified using the single-letter amino acid code.

Mutation	S989P	I1011M	L1021F/H/S	F1534C	D1763Y
Alamogordo	S/S	I/I	L/L	C/C	-
Alamogordo	S/S	I/I	L/L	F/F	D/D
Alamogordo	S/S	I/I	L/L	F/F	D/D
Alamogordo	S/S	I/I	L/L	F/F	D/D
Alamogordo	S/S	I/I	L/L	F/C	D/D
Alamogordo	S/S	I/I	L/L	-	-
Carlsbad	S/S	I/I	L/L	C/C	D/D
Carlsbad	S/S	I/I	L/L	C/C	D/D
Carlsbad	S/S	I/I	L/L	-	-
Carlsbad	S/S	I/I	-	C/C	-
Carlsbad	S/S	I/I	L/L	C/C	D/D
Carlsbad	S/S	I/I	L/L	-	-
Carlsbad	-	-	L/L	-	-
Carlsbad	S/S	I/I	L/L	C/C	D/D
Deming	S/S	I/I	L/L	C/C	D/D
Deming	S/S	I/I	L/L	C/C	D/D
Deming	S/S	I/I	L/L	C/C	D/D
Deming	S/S	I/I	L/L	C/C	D/D
Deming	S/S	I/I	L/L	C/C	D/D
Deming	S/S	I/I	L/L	C/C	D/D
Las Cruces	S/S	I/I	-	F/C	D/D
Las Cruces	S/S	I/I	L/L	C/C	-
Las Cruces	-	-	-	C/C	D/D
Las Cruces	S/S	I/I	L/L	C/C	D/D
Lovington	S/S	I/I	L/L	C/C	D/D
Lovington	-	-	-	F/C	D/D
Lovington	S/S	I/I	-	F/C	D/D
Lovington	S/S	I/I	-	F/C	D/D
Lovington	-	-	-	F/C	D/D
Lovington	S/S	I/I	L/L	C/C	D/D
Roswell	S/S	I/I	L/L	C/C	D/D
Roswell	S/S	I/I	L/L	F/C	D/D
Roswell	S/S	I/I	L/L	C/C	D/D
Roswell	S/S	I/I	L/L	C/C	D/D
Roswell	S/S	I/I	L/L	C/C	D/D
Roswell	S/S	I/I	-	F/C	D/D
Socorro	S/S	I/I	L/L	F/F	D/D
Socorro	S/S	I/I	L/L	F/F	D/D
Socorro	S/S	I/I	-	F/F	D/D
Socorro	-	I/I	-	F/F	D/D
Socorro	S/S	I/I	L/L	F/F	D/D
Socorro	S/S	I/I	L/L	F/F	D/D
Socorro	S/S	I/I	-	F/F	D/D
Sunland	-	-	-	C/C	D/D
Sunland	-	-	-	C/C	D/D
Sunland	S/S	I/I	L/L	C/C	D/D
Sunland	S/S	I/I	L/L	C/C	D/D
Sunland	S/S	I/I	-	C/C	D/D
Sunland	-	-	-	C/C	D/D
Sunland	-	-	-	C/C	D/D
					
Rock (5)	S/S	I/I	L/L	F/F	D/D
Puerto Rico (5)	S/S	I/I	L/L	C/C	D/D

## Discussion

High levels of insecticide resistance in mosquitoes can severely impede vector control programs [15]. During outbreaks of mosquito-borne diseases, insecticide resistance can become a serious threat to public health. Therefore, monitoring levels of resistance to commonly used insecticides in vector populations is paramount for successful pest management during public health emergencies like the recent Zika virus outbreak [53]. The current study characterized, for the first time, insecticide resistance of the mosquito vector *Ae*. *aegypti* across most of its range in New Mexico. The species is largely confined to cities in southern New Mexico, which sit within the Chihuahuan Desert. Its range extends farther north on the eastern edge of the state, and its northeastern range limit is still under investigation. Our unpublished surveys in 2017 and 2018 showed that *Ae*. *aegypti* becomes abundant in all cities in southern New Mexico surveyed to date following the onset of the summer monsoonal rains.

We initiated the current project by requesting information on pesticide use from public vector control programs in New Mexican counties or cities that contain populations of *Ae*. *aegypti*. The most common active ingredients used by our respondents were the pyrethroids permethrin, deltamethrin, etofenprox, and lambda cyhalothrin and, to a lesser degree, the organophosphate acetylcholine-esterase inhibitors malathion and chlorpyrifos. Based on these findings, we decided to proceed with screening for resistance against three pyrethroids and chlorpyrifos. It should be noted that we only received information from public vector control programs and not privately-owned pest control companies or individuals that may have performed mosquito control in or close to the locations we targeted. Therefore, **Table A in S1 File** does not provide a comprehensive list of all of the insecticides to which mosquitoes in an area may have been exposed.

We next established *Aedes aegypti* laboratory strains from field-collected adults from those cities that yielded an adequate number of *Ae*. *aegypti*. Because 135 mosquitoes are required to perform nine replicates of insecticide resistance bottle testing, a minimum of 20 adult F0 females were needed to raise enough F2 mosquitoes for insecticide resistance testing. In order to detect resistance, we used a modified bottle test in which mortality over time in response to high concentrations of target insecticides in a New Mexican *Ae*. *aegypti* population was compared to a well-documented susceptible (Rockefeller) and resistant (Puerto Rico) strain. Our approach is conservative, because “resistance” is defined in the context of a high concentration of insecticide, efficient because mosquitoes need to be monitored for a shorter period of time than a standard CDC bottle assay, affordable because only one concentration of insecticide is used, and highly repeatable because the within-assay comparison among strains minimizes variation due to variation in the concentrations of active ingredients in different batches of insecticides, pipetting errors, and shifts in environmental conditions during the test itself. Further work is needed to compare the accurateness and efficacy of this novel insecticide resistance bioassay to the standard CDC-bottle test and other assays used to measure insecticide resistance.

By our assay, resistance to pyrethroids was widespread in the F2 and F4 generations of strains we established from isolates from New Mexico, with the interesting exception of mosquitoes from Alamogordo, the only city that did not report using pyrethroids for mosquito control. Resistant strains did show considerable variation in survival curves during exposure to permethrin. For example, the mortality curves for the Roswell and Deming strains closely resembled the highly resistant Puerto Rico strain, whereas Las Cruces mosquitoes exhibited a sigmoid mortality curve that lies between the curves for the susceptible and resistant control strains. These patterns suggest that mosquitoes from Las Cruces possess a different set of resistance mechanisms than the Roswell and Deming strains. The mortality curve found for the Lovington strain was double sigmoid, indicating that this strain is a likely a mixture of susceptible and resistant mosquitoes. This hypothesis is supported by the double sigmoid mortality curve that was observed for an even mixture of susceptible Rockefeller and resistant Puerto Rico mosquitoes. The same is true for the Carlsbad strain, but the number of susceptible mosquitoes in this strain is lower. We were not able to perform insecticide resistance testing with deltamethrin, etofenprox, and chlorpyrifos for all strains because limited numbers of mosquitoes were available. In general, we found that strains that were resistant to permethrin were also resistant to other pyrethroids we tested.

We did not find any resistance against the organophosphate chlorpyrifos in our tests. This confirms findings from an earlier study showing that pyrethroid resistance and resistance to chlorpyrifos are not always correlated [54]. Chlorpyrifos could therefore be considered a viable alternative to pyrethroids; however, the use of chlorpyrifos as a public health insecticide is controversial because of its adverse effects on prenatal human development [55]. Also, chlorpyrifos resistance has been found in wild populations of *Culex* mosquitoes [56] and it has been experimentally evolved in laboratory experiments [57].

It must be noted that the laboratory strains we established for different cities do not necessarily reflect resistance levels in *Aedes aegypti* populations in these cities. A more extensive sampling regime would be necessary to study how widespread resistance is geographically and over time.

To explore the genetic basis for pyrethroid resistance in New Mexican mosquitoes, we sequenced fragments of the *para* gene of 50 mosquitoes from different cities in New Mexico and showed that only one known kdr mutation was present–F1534C. This specific mutation is notorious around the world where pyrethroids have been used to control *Ae*. *aegypti* [36, 58, 59]. It has been suggested that this particular mutation confers no or very little fitness reduction [60, 61]. This lack in fitness cost explains the fact that insecticide resistance can develop quickly in *Ae*. *aegypti* and it can persist for years, even after the relevant active ingredients are no longer used [62]. The high level of kdr mutations within the mosquitoes we analyzed suggests that alternatives to pyrethroids should be considered for mosquito control in New Mexico.

Two significant caveats pertain to this study. First, we used F2 and F4 mosquitoes for the resistance testing because we were not able to procure enough F0 or F1 mosquitoes from the field. This strategy has been used before [63, 64], however, starting a population from low numbers of individuals bears the risk that their offspring don’t represent all genotypes and phenotypes present in the field populations. In order to preserve field levels of resistance, we used laboratory generations not higher than F4. However, we did capture a wide geographic swath of populations; the two most distant cities in our study, Deming and Lovington, are separated by approximately 300 miles. Second, a relatively low number of mosquitoes from different cities in New Mexico was analyzed genetically (between 4 and 8 individuals), preventing us from correlating the levels of pyrethroid resistance with frequencies of kdr mutations in individual field populations. Nonetheless, the finding that the kdr mutation is common in New Mexico is intriguing and should motivate more extensive studies.

## Conclusion

*Ae*. *aegypti* from New Mexico showed high levels of pyrethroid resistance and high incidence of the kdr mutation F1534C in the para gene. All strains that we tested were still susceptible to the organophosphate chlorpyrifos. Based on the results of our study, we recommend a statewide resistance monitoring and management program to aid the control of *Ae*. *aegypti* and other disease-transmitting mosquitoes that threaten public health. Our results also stress the importance of developing alternative approaches to mosquito control like sterile-insect-technique [65, 66] or the use of *Wolbachia*, [67, 68].

## Supporting information

S1 File Contains supporting Figs A-J and supporting Tables A & B. (PDF)
