# Supplementary material for: Widespread insecticide resistance in Aedes aegypti L. from New Mexico, U.S.A
Source: PLoS One. 2019 Feb 22;14(2):e0212693. doi: 10.1371/journal.pone.0212693 (PMC6386485; doi:10.1371/journal.pone.0212693)
Supplement: S1 File — (PDF) [file pone.0212693.s001.pdf]

Deltamethrin Resistance Tests: Alamogordo strain

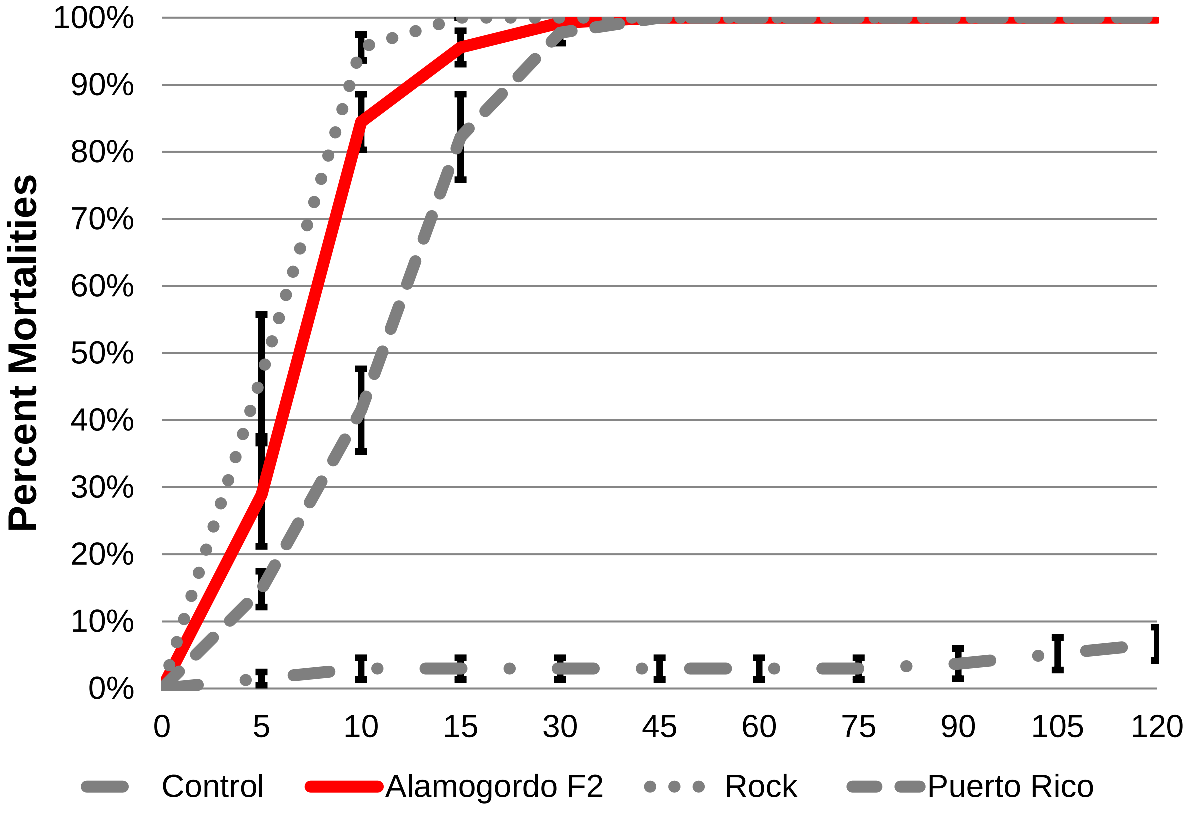

Figure A. Deltamethrin IR Bottle Test Results: 10.5 ug/bottle. Shown are means and standard error bars for different groups of mosquitoes. Rock is considered a pyrethroid-sensitive strain. Puerto Rico is considered a pyrethroid-resistant strain. The control was treated with acetone only.

Results- The control (Acetone) mortality curve was significantly different (P<0.0001) from the curves for all other treatments with little to no mortalities. Kaplan Meier statistical analysis confirmed that there was a significant difference between the the curves plotted for the deltamethrin-exposed Rock Strain and the Alamogordo Strain (P<.0001). The curve plotted for the Puerto Rico Strain (a known resistant strain) is significantly different than that plotted for the Alamogordo Strain (P<.0001). The curves plotted for the Puerto Rico strain and the Rock strain were also significantly different from each other (P<.0001).

Conclusion- Although we found small but statistical significant differences in the survival curve of the ROCK control and the curve plotted for mosquitoes from Alamogordo, the comparison with the curve plotted for the resistant Puerto Rico strain suggests that the Alamogordo strain is sensitive to deltamethrin.

Deltamethrin Resistance Tests: Las Cruces strain

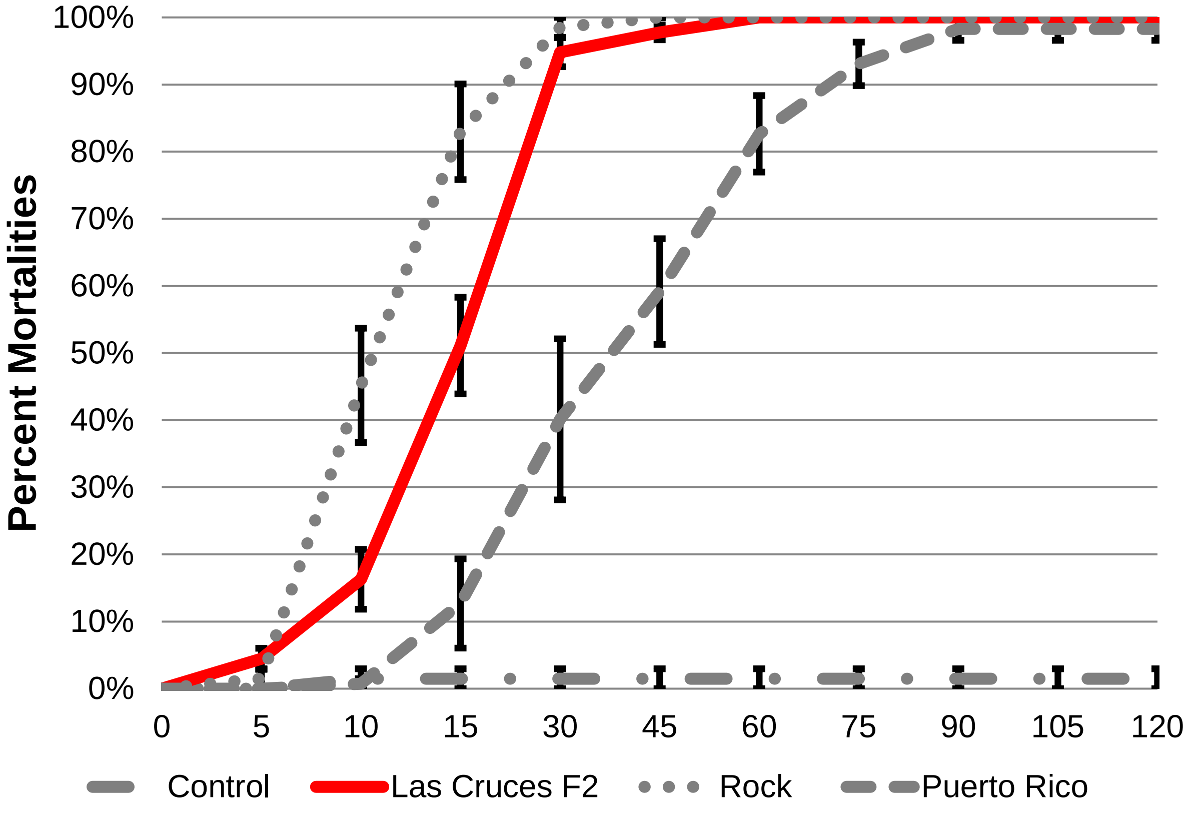

Figure B. Deltamethrin IR Bottle Test Results: 10.5 ug/bottle. Shown are means and standard error bars for different groups of mosquitoes. Rock is considered a pyrethroid-sensitive strain. Puerto Rico is considered a pyrethroid-resistant strain. The control was treated with acetone only.

Results- The control (Acetone) mortality curve was significantly different ($P<0.0001$) from the curves for all other treatments with little to no mortalities. Kaplan Meier statistical analysis confirmed that there was a significant difference between the curves plotted for the deltamethrin-exposed Rock Strain and the Las Cruces Strain ($P<.0001$). The curve plotted for the Puerto Rico Strain (a known resistant strain) is significantly different than that plotted for the Las Cruces Strain ($P<.0001$). The curves plotted for the Puerto Rico strain and the Rock strain were also significantly different from each other ($P<.0001$).

Conclusion- the Las Cruces *Aedes aegypti* strain showed resistance toward deltamethrin.

Deltamethrin Resistance Tests: Carlsbad strain

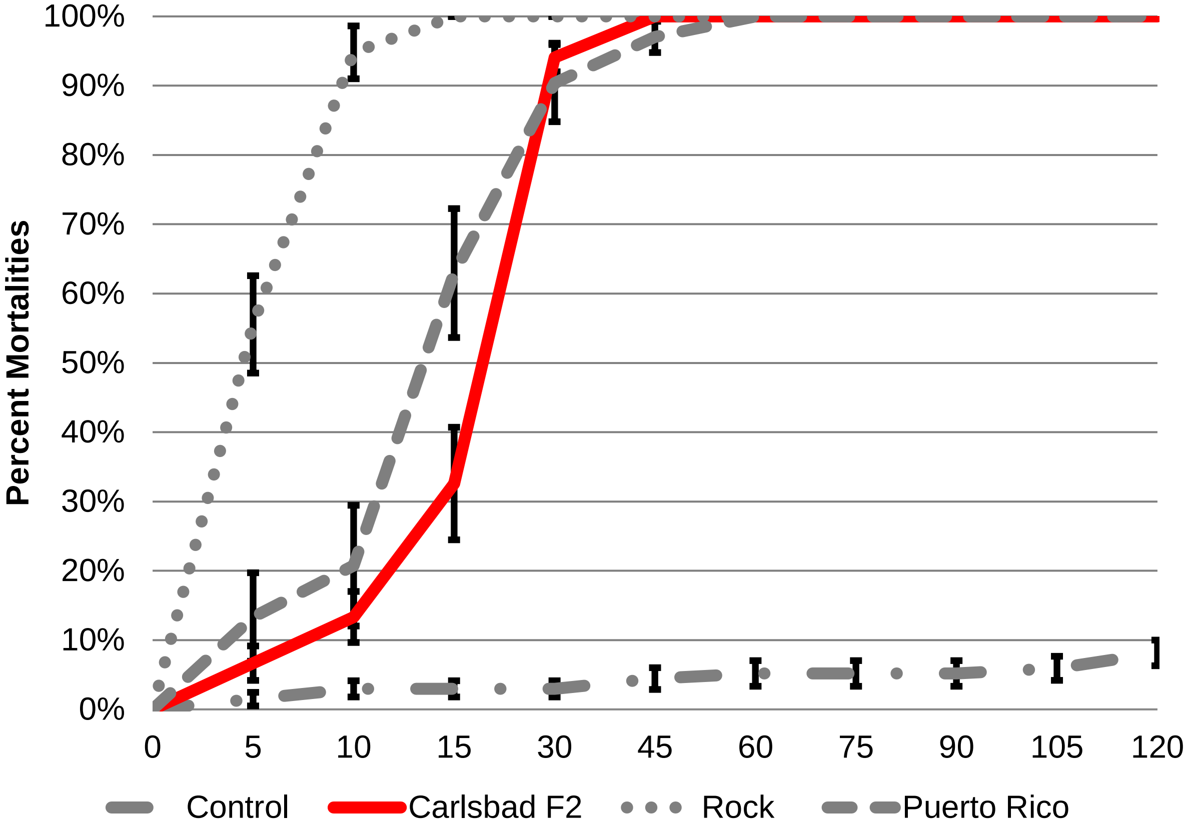

Figure C. Deltamethrin IR Bottle Test Results: 10.5 ug/bottle. Shown are means and standard error bars for different groups of mosquitoes. Rock is considered a pyrethroid-sensitive strain. Puerto Rico is considered a pyrethroid-resistant strain. The control was treated with acetone only.

Results- The control (Acetone) mortality curve was significantly different (P<0.0001) from the curves for all other treatments with little to no mortalities. Kaplan Meier statistical analysis confirmed that there was a significant difference between the the curves plotted for the deltamethrin-exposed Rock Strain and the Carlsbad Strain (P<.0001). The curve plotted for the Puerto Rico Strain (a known resistant strain) is significantly different than that plotted for the Carlsbad Strain (P<.003). The curves plotted for the Puerto Rico strain and the Rock strain were also significantly different from each other (P<.0001).

Conclusion- The Carlsbad *Aedes aegypti* strain shows resistance to deltamethrin.

Deltamethrin Resistance Tests: Roswell strain

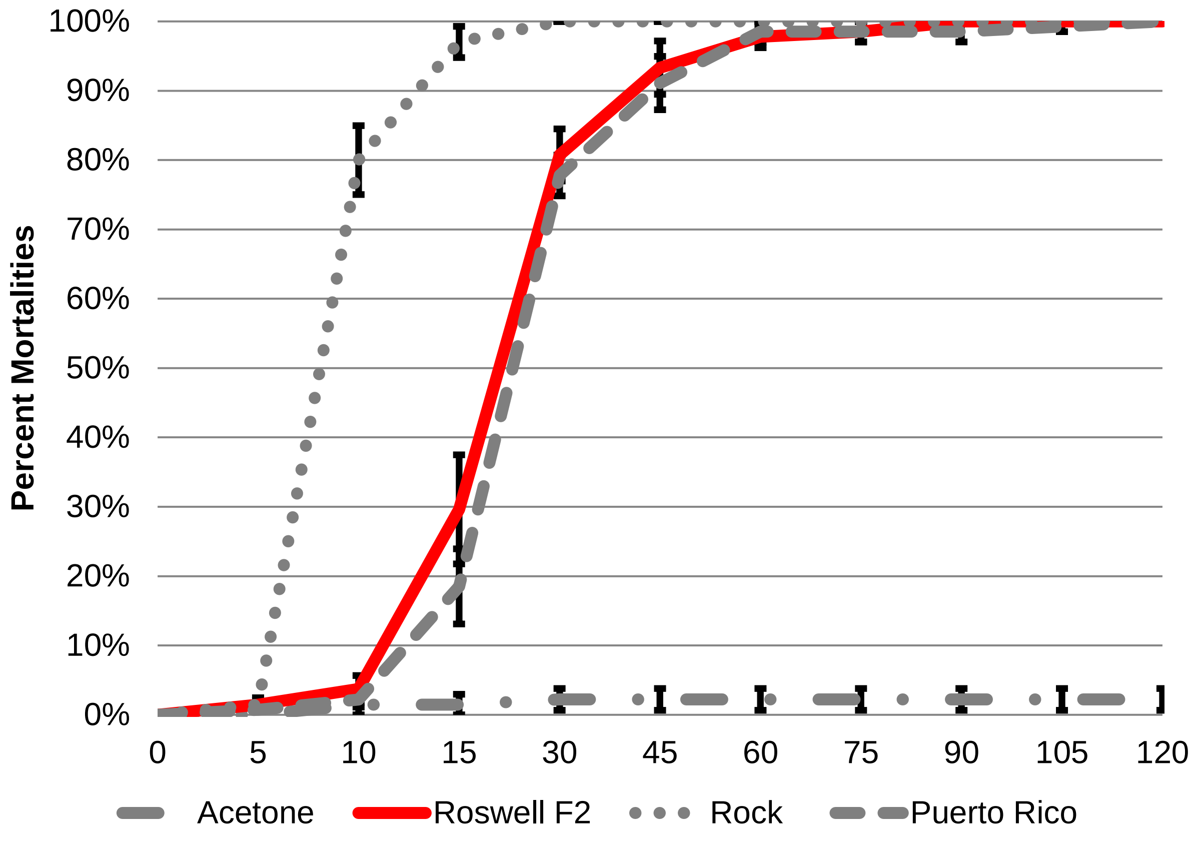

Figure D. Deltamethrin IR Bottle Test Results: 10.5 ug/bottle. Shown are means and standard error bars for different groups of mosquitoes. Rock is considered a pyrethroid-sensitive strain. Puerto Rico is considered a pyrethroid-resistant strain. The control was treated with acetone only.

Results- The control (Acetone) mortality curve was significantly different (P<0.0001) from the curves for all other treatments with little to no mortalities. Kaplan Meier statistical analysis confirmed that there was a significant difference between the curves plotted for the deltamethrin-exposed Rock Strain and the Roswell Strain (P<.0001). The curve plotted for the Puerto Rico Strain (a known resistant strain) is not significantly different than that plotted for the Roswell Strain (P=.091). The curves plotted for the Puerto Rico strain and the Rock strain were also significantly different from each other (P<.0001).

Conclusion- The Roswell *Aedes aegypti* strain shows resistance towards deltamethrin.

Etofenprox Resistance Tests: Roswell strain

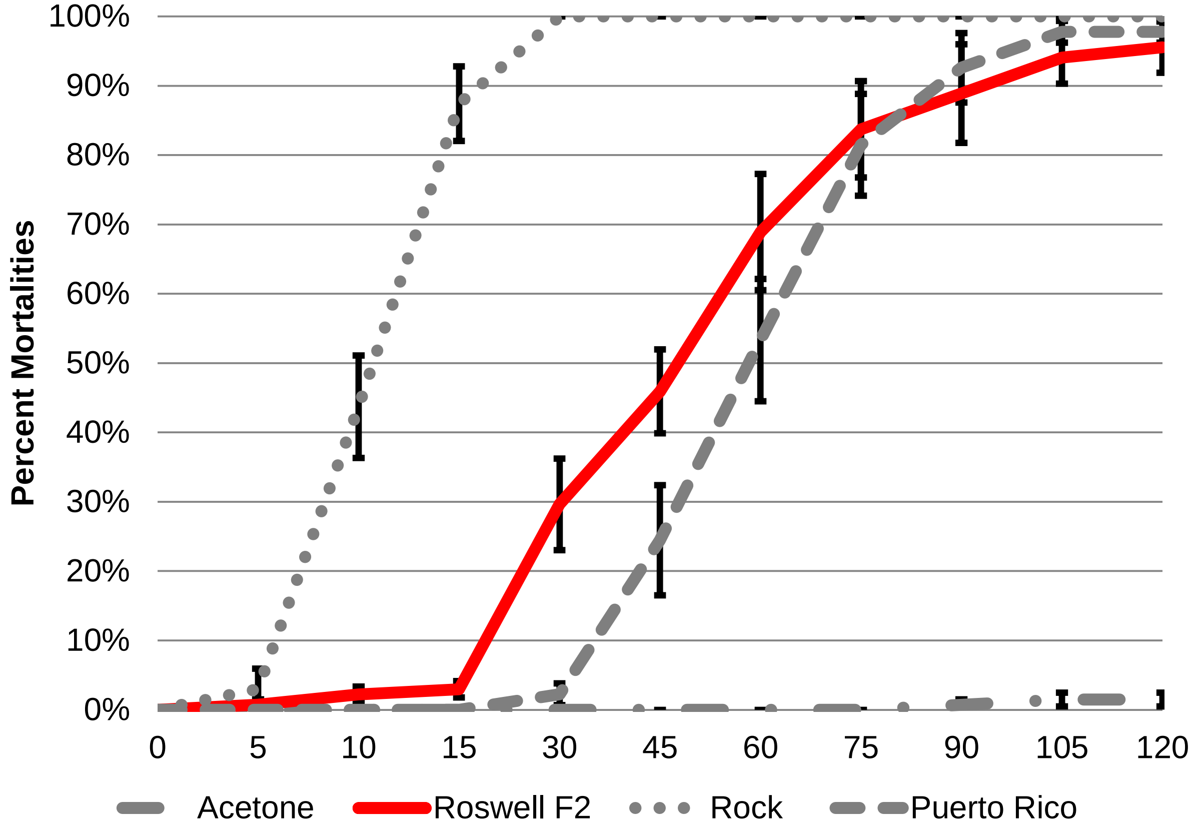

Figure E. Etofenprox IR Bottle Test Results: 37.5 ug/bottle. Shown are means and standard error bars for different groups of mosquitoes. Rock is considered a pyrethroid-sensitive strain. Puerto Rico is considered a pyrethroid-resistant strain. The control was treated with acetone only.

Results- The control (Acetone) mortality curve was significantly different (P<0.0001) from the curves for all other treatments with little to no mortalities. Kaplan Meier statistical analysis confirmed that there was a significant difference between the curves plotted for the etofenprox-exposed Rock Strain and the Roswell Strain (P<.0001). The curve plotted for the Puerto Rico Strain (a known resistant strain) is significantly different than that plotted for the Roswell Strain (P=.034). The curves plotted for the Puerto Rico strain and the Rock strain were also significantly different from each other (P<.0001).

Conclusion- The Roswell *Aedes aegypti* strain shows resistance towards etofenprox.

Etofenprox Resistance Tests: Las Cruces strain

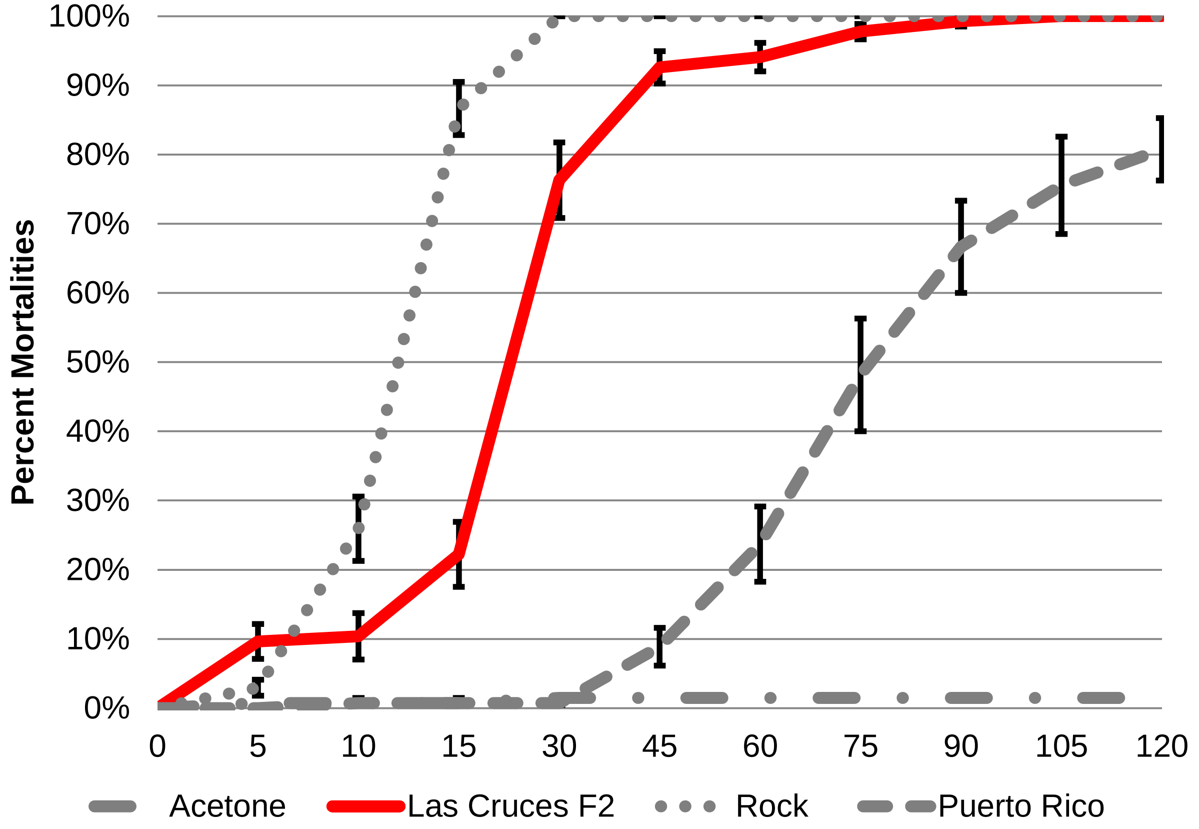

Figure F. Etofenprox IR Bottle Test Results: 37.5 ug/bottle. Shown are means and standard error bars for different groups of mosquitoes. Rock is considered a pyrethroid-sensitive strain. Puerto Rico is considered a pyrethroid-resistant strain. The control was treated with acetone only.

Results- The control (Acetone) mortality curve was significantly different (P<0.0001) from the curves for all other treatments with little to no mortalities. Kaplan Meier statistical analysis confirmed that there was a significant difference between the curves plotted for the etofenprox-exposed Rock Strain and the Las Cruces Strain (P<.0001). The curve plotted for the Puerto Rico Strain (a known resistant strain) is significantly different than that plotted for the Las Cruces Strain (P<.0001). The curves plotted for the Puerto Rico strain and the Rock strain were also significantly different from each other (P<.0001).

Conclusion- The Las Cruces *Aedes aegypti* strain shows resistance towards etofenprox.

Chlorpyrifos Resistance Tests: Las Cruces strain

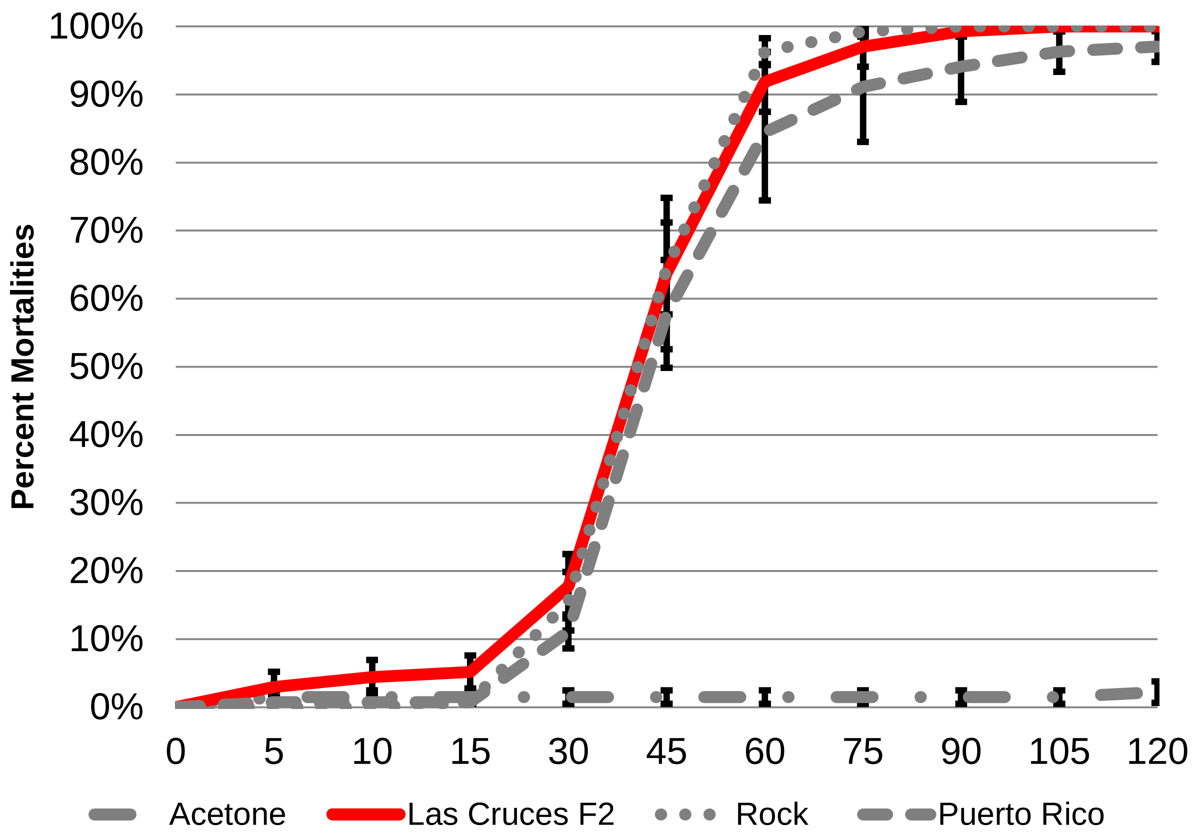

Figure G. Chlorpyrifos IR Bottle Test Results: 40 ug/bottle. Shown are means and standard error bars for different groups of mosquitoes. Rock is considered a pyrethroid-sensitive strain. Puerto Rico is considered a pyrethroid-resistant strain. The control was treated with acetone only.

Results- The control (Acetone) mortality curve was significantly different (P<0.0001) from the curves for all other treatments with little to no mortalities. Kaplan Meier statistical analysis confirmed that there was no significant difference between the curves plotted for the chlorpyrifos-exposed Rock Strain and the Las Cruces Strain (P=.589). The curve plotted for the Puerto Rico Strain (a known resistant strain) is significantly different than that plotted for the Las Cruces Strain (P=.014). The curves plotted for the Puerto Rico strain and the Rock strain were also significantly different from each other (P=.005).

Conclusion- The Las Cruces *Aedes aegypti* strain is sensitive towards chlorpyrifos.

Chlorpyrifos Resistance Tests: Alamogordo strain

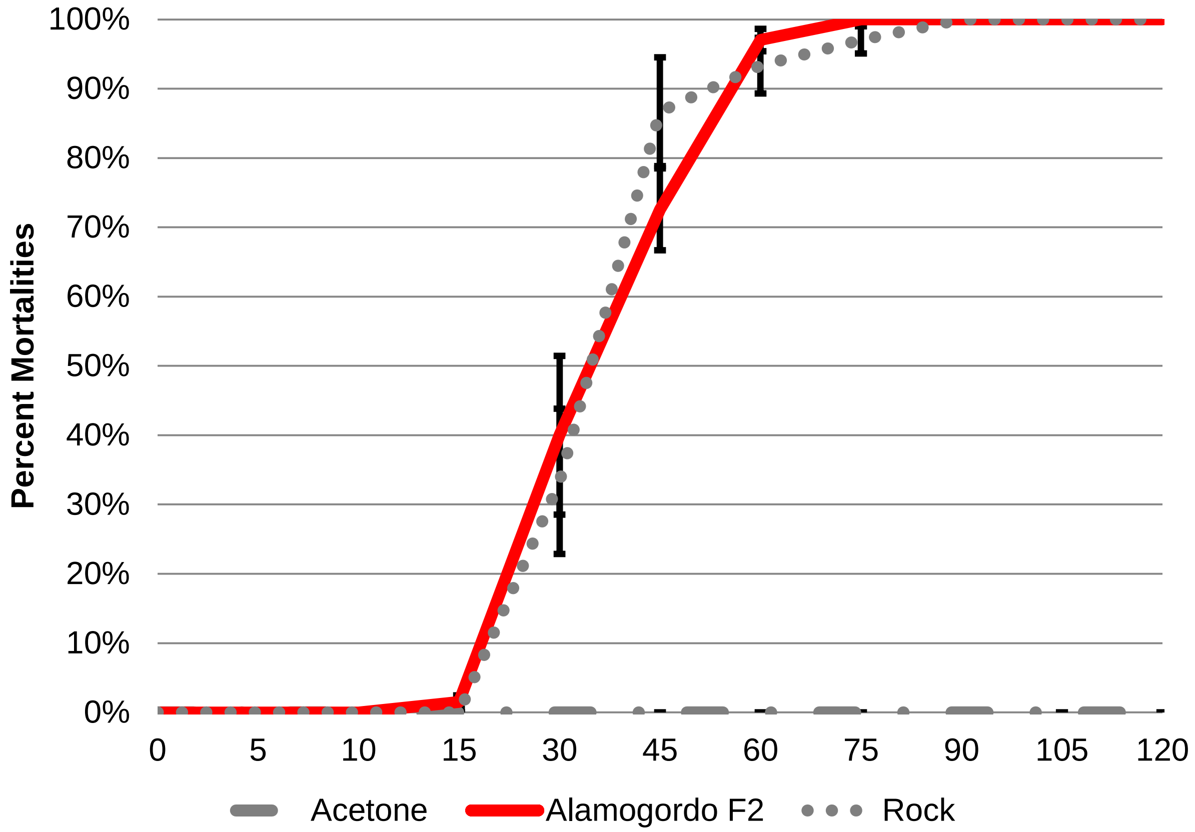

Figure H. Chlorpyrifos IR Bottle Test Results: 40 ug/bottle. Shown are means and standard error bars for different groups of mosquitoes. Rock is considered a pyrethroid-sensitive strain. The control was treated with acetone only.

Results- The control (Acetone) mortality curve was significantly different ($P<0.0001$) from the curves for all other treatments with little to no mortalities. Kaplan Meier statistical analysis confirmed that there was not a significant difference between the curves plotted for the chlorpyrifos-exposed Rock Strain and the Alamogordo Strain ($P=.984$).

Conclusion- The Alamogordo *Aedes aegypti* strain is sensitive to chlorpyrifos.

Chlorpyrifos Resistance Tests: Carlsbad strain

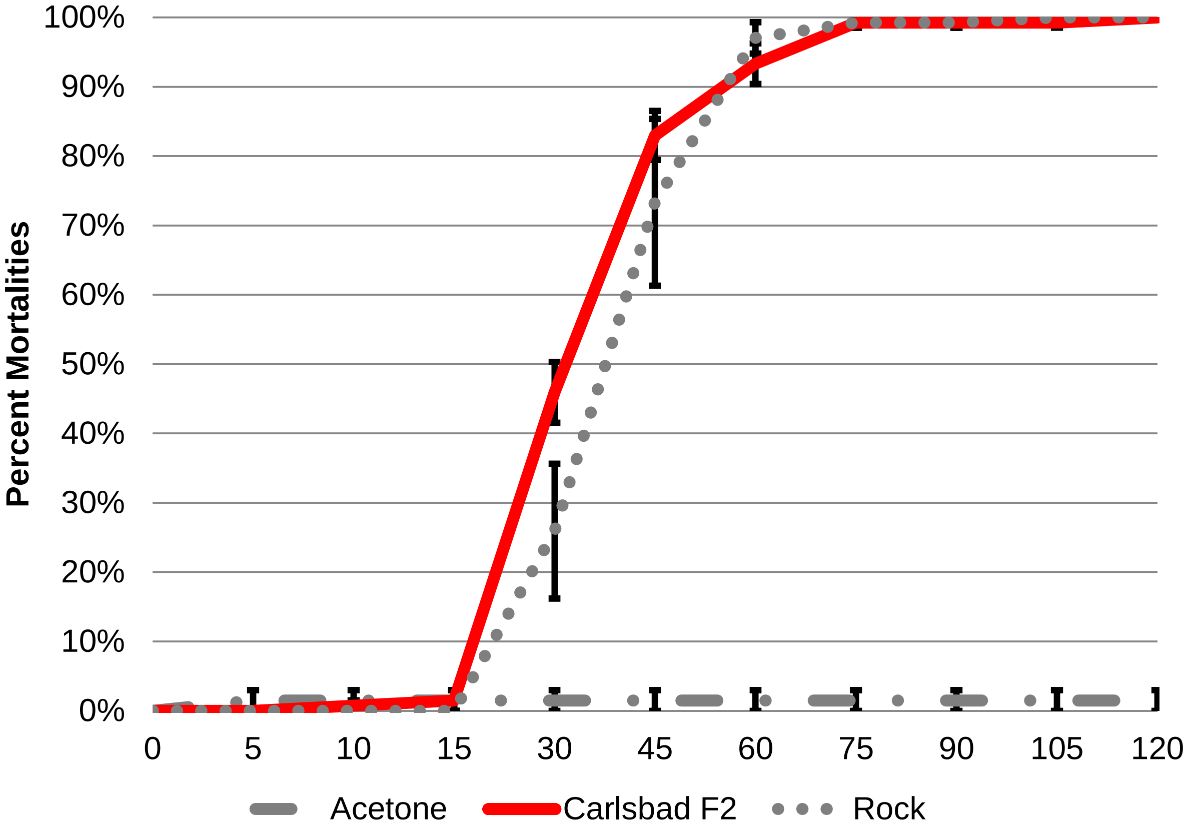

Figure I. Chlorpyrifos IR Bottle Test Results: 40 ug/bottle. Shown are means and standard error bars for different groups of mosquitoes. Rock is considered a pyrethroid-sensitive strain. The control was treated with acetone only.

Results- The control (Acetone) mortality curve was significantly different ($P<0.0001$) from the curves for all other treatments with little to no mortalities. Kaplan Meier statistical analysis confirmed that there was a significant difference between the curves plotted for the chlorpyrifos-exposed Rock Strain and the Carlsbad Strain ($P=.028$).

Conclusion- The Carlsbad *Aedes aegypti* strain shows sensitivity towards chlorpyrifos.

Chlorpyrifos Resistance Tests: Roswell strain

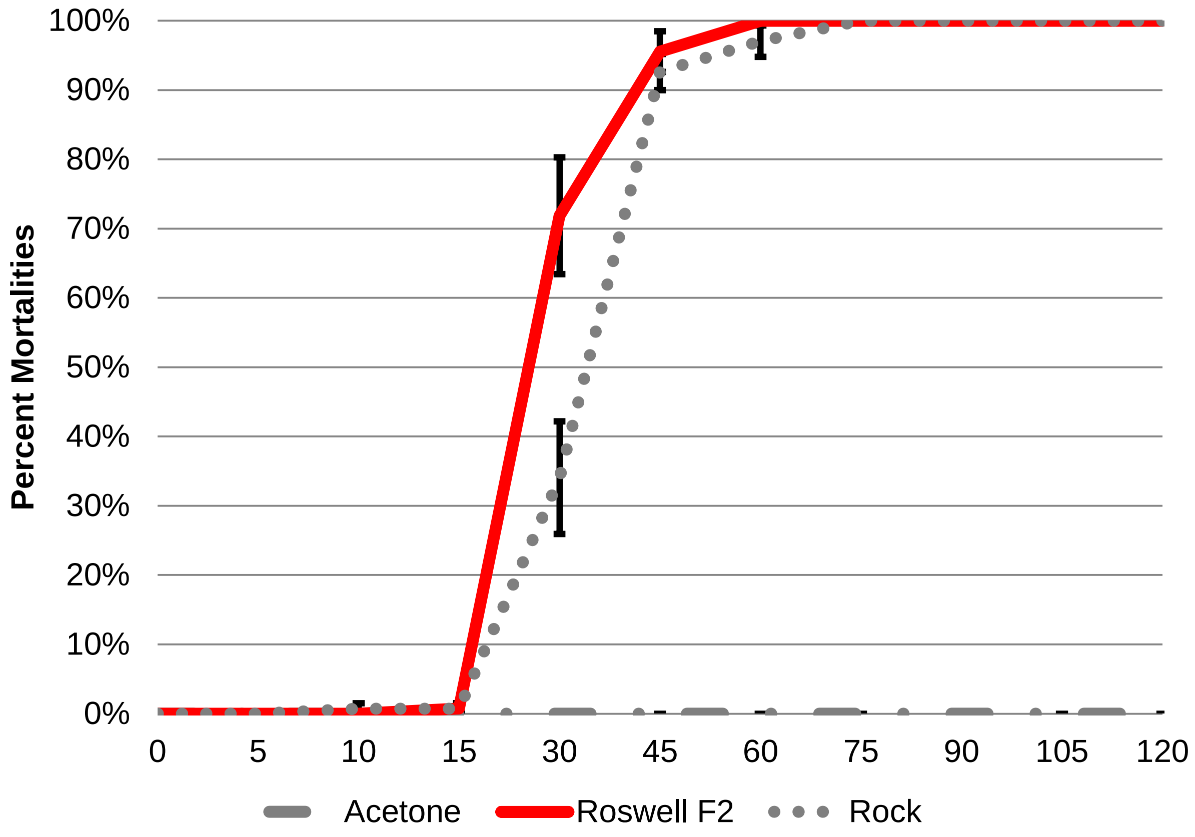

Figure J. Chlorpyrifos IR Bottle Test Results: 40 ug/bottle. Shown are means and standard error bars for different groups of mosquitoes. Rock is considered a pyrethroid-sensitive strain. The control was treated with acetone only.

Results- The control (Acetone) mortality curve was significantly different (P<0.0001) from the curves for all other treatments with little to no mortalities. Kaplan Meier statistical analysis confirmed that there was a significant difference between the the curves plotted for the chlorpyrifos-exposed Rock Strain and the Roswell Strain (P<.0001).

Conclusion- The Roswell *Aedes aegypti* strain is sensitive to chlorpyrifos.

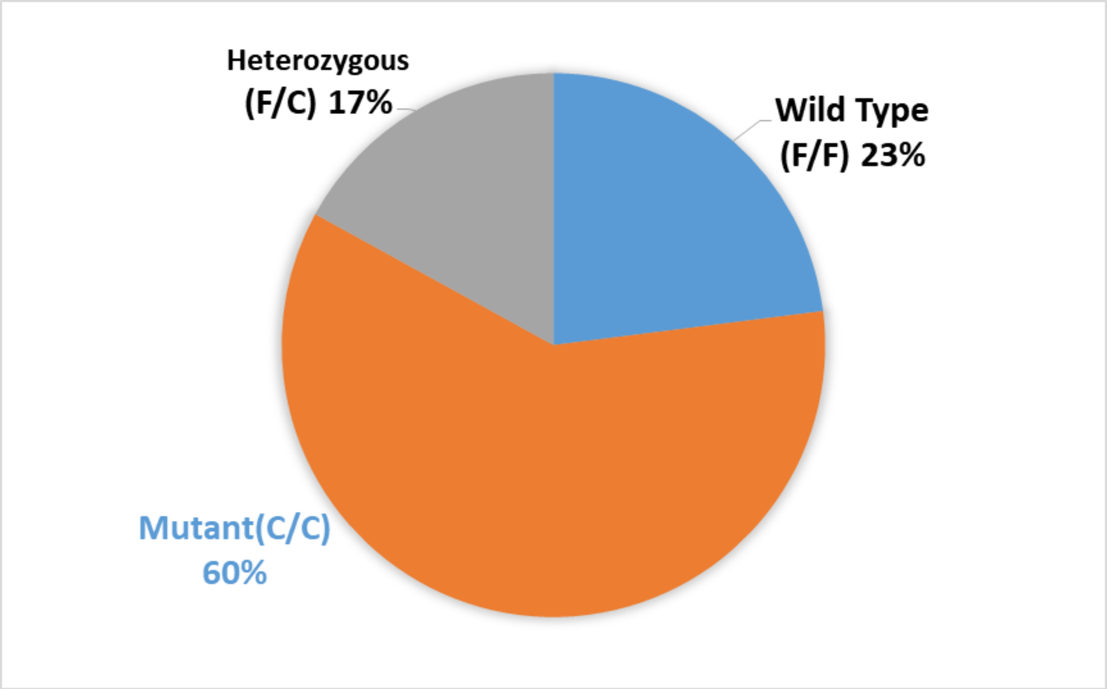

Figure K: kdr position 1534 Genotypes in 50 mosquitoes from New Mexico. The figure shows the percentage of heterozygous and homozygous mosquitoes. The resistant mutants are shown in orange, wild type in blue, and heterozygous in grey.

```
[illegible]09_aegypt        MTEDSDSISEEERSLFRPPFTRESLAAIERRIADAEAKQRELEKKRAEGETGFGRKKKKKE  60
[illegible]65908_Musca      MTEDSDSISEEERSLFRPPFTRESLLQIEQRIAEHEKQ-KELERKRA---------AEGEQ  50
                            ************************  **:***: * : :***:***          : ::

NC_035109_aegypt            IRYDDEDEDEGPQPDSTLEQGVPIPVRMQGSFPPELASTPLEDIDSYYANQRTFVVVSKG  120
NW_004765908_Musca          IRYDDEDEDEGPQPDPTLEQGVPIPVRMQGSFPPELASTPLEDIDPFYSNVLTFVVISKG  110
                            *************** ***************************** :*:*  ****:***

NC_035109_aegypt            KDIFRFSATNALYVLDPFNPIRRVAIYILVHPLFSFFIITTILTNCILMIMPSTPTVEST  180
NW_004765908_Musca          KDIFRFSASKAMWLLDPFNPIRRVAIYILVHPLFSLFIITTILTNCILMIMPTTPTVEST  170
                            ********::*:::********************:****************:*******

NC_035109_aegypt            EVIFTGIYTFESAVKVMARGFILQPFTYLRDAWNWLDFVVIALAYVTMGIDLGNLAALRT  240
NW_004765908_Musca          EVIFTGIYTFESAVKVMARGFILCPFTYLRDAWNWLDFVVIALAYVTMGIDLGNLAALRT  230
                            *********************** ************************************

NC_035109_aegypt            FRVLRALKTVAIVPGLKTIVGAVIESVKNLRDVIILTMFSLSVFALMGLQIYMGVLTQKC  300
NW_004765908_Musca          FRVLRALKTVAIVPGLKTIVGAVIESVKNLRDVIILTMFSLSVFALMGLQIYMGVLTQKC  290
                            ************************************************************

NC_035109_aegypt            IREFPMDGSWGNLSDENWERFNNNDSNWYFSETGD-TPLCGNSSGAGQCEEGYICLQGYG  359
NW_004765908_Musca          IKRFPLDGSWGNLTDENWFLHNSNSSNWFTENDGESYPVCGNVSGAGQCGEDYVCLQGFG  350
                            *:.**:*******:****   .*.*.***:  .: *:   *:*** ****** *.*:**   419

NC_035109_aegypt            DNPNYGYTSFDTFGWAFLSAFRLMTQDYWENLYQLVLRSAGPWHMLFFIVIIFLGSFYLV  419
NW_004765908_Musca          PNPNYDYTSFDSFGWAFLSAFRLMTQDFWEDLYQHVLQAAGPWHMLFFIVIIFLGSFYLV  410
                             ****.*****:***************:**:*** **::*****************   410

NC_035109_aegypt            NLILAIVAMSYDELQKRAEEEEEAAEEEALREAEEAAAAKAAKLEAQAAA----------  468
NW_004765908_Musca          NLILAIVAMSYDELQKKAEEEEEAAEEEAIREAEEAAAAKAAKLEERANVAAQAAQDAADA  470
                            ****************:***********:*************** :*  .

NC_035109_aegypt            AAAAANPEIAKSPSDFSCHSYELFVNQEKGNDDNNKEKMSIRSEGLESVSEITRTTAPTA  528
NW_004765908_Musca          AAAALHPEMAKSP-TYSCISYELFVGGEKGNDDNNKEKMSIRSVEVESESVSVIQRQPAP  529
                            ****  :**:****   :** ******.  ****************   :** *   .    *:

NC_035109_aegypt            TAAGTAKARKVSAGVAAFQKASLSLPGPSFPNLRRGSRGSHQFTIRNGRGRFVGVPGSDRK  588
NW_004765908_Musca          TTAPATKVR-------KVSTTSLSLPGPSFPNLRRGSRSSHKYTIRNGRGRF-GIPGSDRK  581
                            *:*  ::*.*        ...:****************.**::********* *:******

NC_035109_aegypt            PLVLSTYLDAQEHLPYADDSNAVTPMSEENGAIIVPVYYANLGSRHSSYTSHQSRISYTS  648
NW_004765908_Musca          PLVLQTYQDAQQHLPYADDSNAVTPMSEENGAIIVPAYYCNLGSRHSSYTSHQSRISYTS  641
                            ****.** ***:************************.**.********************

NC_035109_aegypt            HGDLLGGMT--------KESRLRNRSARNTNHSIVPPPNMSGPNMSYVDSNHKGQRDFDM  700
NW_004765908_Musca          HGDLLGGMAAMGASTMTKESKLRSRNTRNQSIGAATNGGSSTAGGGYPDANHKEQRDYEM  701
                            ********:        ***:**.*.:**  .  .  .    .  *    . .* *:*** ***::*

NC_035109_aegypt            SQDCTDEAGKIKHNDNPFIEPSQTQTVVDMKDVMVLNDIIEQAAGRHSRASDHGVSVYYF  760
NW_004765908_Musca          GQDYTDEAGKIKHHDNPFIEPVQTQTVVDMKDVMVLNDIIEQAAGRHSRASERG------  755
                            .** *********:******* *****************************::*

NC_035109_aegypt            PTEDDDDEDGPTFKDKALEFTMRMIDVFCVWDCCWVWLKFQEWVAFIVFDPFVELFITLCI  820
NW_004765908_Musca          --EDDDEDGPTFKDIALEYILKGIEIFCVWDCCWVWLKFQEWVSFIVFDPFVELFITLCI  813
                              ************ ***: :: *::*****************:****************

NC_035109_aegypt            VVNTLFMALDHHDMDPDMERALKSGNYFFTATFAIEATMKLIAMSPKYYFQEGWNIFDFI  880
NW_004765908_Musca          VVNTMFMAMDHHDMNPELEKVLKSGNYFFTATFAIEASMKLMAMSPKYYFQEGWNIFDFI  873
                            ****:***:*****:*::*:.****************:***:****   930   *******

NC_035109_aegypt            IVALSLLELGLEGVQGLSVLRSFRLLRVFKLAKSWPTLNLLISIMGRTMGALGNLTFVLC  940
NW_004765908_Musca          IVALSLLELGLEGVQGLSVLRSFRLLRVFKLAKSWPTLNLLISIMGRTMGALGNLTFVLC  933
                            **********************************************   923   *******

                                                                       989    996
NC_035109_aegypt            IIIFIFAVMGMQLFGKNYIDNVDRFPDKDLPRWNFTDFMHSFMIVFRVLCGEWIESMWDC  1000
NW_004765908_Musca          IIIFIFAVMGMQLFGKNYIDHKDRFKDHELPRWNFTDFMHSFMIVFRVLCGEWIESMWDC  993
                            ********************: *** *::*****************   982    989
```

```
                                              1018  1023
NC_035109_aegypt      MLVGDVSCIPFFLATVVIGNLVGLNLFLALLLSNFGSSSL SAPTADNETNKIAEAFNRIS  1060
NW_004765908_Musca    MYVGDVSCIPFFLATVVIGNLVVLNLFLALLLSNFGSSSLSAPTADNDTNKIAEAFNRIA  1053
                      * ***********  1011  1016  ********************:***********:
                                              
NC_035109_aegypt      RFSNWIKSNIANALKFVKNKLTSQIASVQPAGEQHNHLSWIWNEGKGVCPCISAEHGENE  1120
NW_004765908_Musca    RFKNWVKRNIADCFKLIRNKLTNQISDQ-----------------------PSEHGDNE  1089
                      **.**:* ***:.:*:::****.**:.                        :***:**

NC_035109_aegypt      LELTPDDILADGLLKKGVKEHNQLEVAIGDGMEFTIHGDLKNKGKKNKQLMNNSKVIGNS  1180
NW_004765908_Musca    LELGHDEIMGDGLIKKGMKGETQLEVAIGDGMEFTIHGDMKNNKPKKSKFINNTTMIGNS  1149
                      ***  *:*:.***:***:*  ..*****************:**:  *:.:::**:.:****

NC_035109_aegypt      ISNHQDNKLEHELNHRGMSLQDDDTASIKSYGSHKNRPFKDESHKGSAETMEGEEKRDVS  1240
NW_004765908_Musca    I-NHQDNRLEHELNHRGLSIQDDDTASINSYGSHKNRPFKDESHKGSAETIEGEEKRDVS  1208
                      * *****:*********:*:********:*********************:*********

NC_035109_aegypt      KEDLGIDEELDDECDGEEGPLDGELIIHADE-DEVIEDSPADCCPDNCYKKFPVLAGDDD  1299
NW_004765908_Musca    KEDLGLDEELDEEAEGDEGQLDGDIIIHAQNDDEIIDDYPADCFPDSYYKKFPILAGDED  1268
                      *****:*****:*.:*:** ***::****:: **:*:* **** **. *****:****:*

NC_035109_aegypt      APFWQGWANLRLKTFQLIENKYFETAVITMILLSSLALALEDVHLPHRPILQDVLYYMDR  1359
NW_004765908_Musca    SPFWQGWGNLRLKTFQLIENKYFETAVITMILMSSLALALEDVHLPDRPVMQDILYYMDR  1328
                      :******.************************:*************.**::**:******

NC_035109_aegypt      IFTVIFFLEMLIKWLALGFRVYFTNAWCWLDFIIVMVSLINFVASLCGAGGIQAFKTMRT  1419
NW_004765908_Musca    IFTVIFFLEMLIKWLALGFKVYFTNAWCWLDFVIVMLSLINLVAVWSGLNDIAVFRSMRT  1388
                      *******************:************:***:****:**   .*  ..*  .*::***

NC_035109_aegypt      LRALRPLRAMSRMQGMRVVVNALVQAIPSIFNVLLVCLIFWLIFAIMGVQLFAGKYFKCV  1479
NW_004765908_Musca    LRALRPLRAVSRWEGMKVVVNALVQAIPSIFNVLLVCLIFWLIFAIMGVQLFAGKYFKCK  1448
                      *********:**  :**:******************************************

NC_035109_aegypt      DKNKTTLSHEIIPDVNACVAENYTWENSPMNFDHVGKAYLCLFQVATFKGWIQIMNDAID  1539
NW_004765908_Musca    DGNDTVLSHEIIPNRNACKSENYTWENSAMNFDHVGNAYLCLFQVATFKGWIQIMNDAID  1508
                      * *.*.**  1551  ** 1565  ***** *******:***********************
                                            
NC_035109_aegypt      SREVGKQPIRETNIYMYLYFVFFIIFGSFFTLNLFIGVIIDNFNEQKKKAGGSLEMFMTE  1599
NW_004765908_Musca    SREVDKQPIRETNIYMYLYFVFFIIFGSFFTLNLFIGVIIDNFNEQKKKAGGSLEMFMTE  1568
                      ****.***  1520  * 1534  **************************************
                                            
NC_035109_aegypt      DQKKYYNAMKKMGSKKPLKAIPRPRWRPQAIVFEIVTNKKFDMIIMLFIGFNMLTMTLDH  1659
NW_004765908_Musca    DQKKYYNAMKKMGSKKPLKAIPRPRWRPQAIVFEIVTDKKFDIIIMLFIGLNMFTMTLDR  1628
                      *************************************:****:*******:**:*****:

NC_035109_aegypt      YKQTDTFSAVLDYLNMIFICIFSSECLMKIFALRYHYFIEPWNLFDFVVVILSILGLVLS  1719
NW_004765908_Musca    YDASEAYNNVLDKLNGIFVVIFSGECLLKIFALRYHYFKEPWNLFDVVVVILSILGLVLS  1688
                      *. ::::. *** ** **: ***.***:********** *******.**************

NC_035109_aegypt      DLIEKYFVSPTLLRVVRVAKVGRVLRLVKGAKGIRTLLFALAMSLPALFNICLLLFLVMF  1779
NW_004765908_Musca    DIIEKYFVSPTLLRVVRVAKVGRVLRLVKGAKGIRTLLFALAMSLPALFNICLLLFLVMF  1748
                      *:*********  1794  *****************************************
                                              
NC_035109_aegypt      IFAIFGMSFFMHVKYKSGLDDVYNFKTFGQSMILLFQMSTSAGWDGVLDGIINEDECLPP  1839
NW_004765908_Musca    IFAIFGMSFFMHVKEKSGINAVYNFKTFGQSMILLFQMSTSAGWDGVLDAIINEEDCDPP  1808
                      ***********  1763  :: ****************************.****::* **
                                              
NC_035109_aegypt      DNDKGYPGNCGSATIGITYLLAYLVISFLIVINMYIAVILENYSQATEDVQEGLTDDDYD  1899
NW_004765908_Musca    DNDKGYPGNCGSATVGITFLLSYLVISFLIVINMYIAVILENYSQATEDVQEGLTDDDYD  1868
                      **************:***:**:**************************************

NC_035109_aegypt      MYYEIWQQFDPDGTQYIRYDQLSDFLDVLEPPLQIHKPNKYKIISMDIPICRGDMMFCVD  1959
NW_004765908_Musca    MYYEIWQQFDPEGTQYIRYDQLSEFLDVLEPPLQIHKPNKYKIISMDMPICRGDMMYCVD  1928
                      ***********:***********:***********************:********:***

NC_035109_aegypt      ILDALTKDFFARKGNPIEETAELGEVQARPDEVGYEPVSSTLWRQREEYCARVIQHAWRK  2019
NW_004765908_Musca    ILDALTKDFFARKGNPIEETGEIGEIAARPDTEGYDPVSSTLWRQREEYCAKLIQNAWRR  1988
                      ********************.*:**: ****  **:****************::**:***:

NC_035109_aegypt      HKERQAGGGGGDDTDADACDNDDGDDGGGGAGDGGSAGGGGVT-SPGVGSGSIVGGGTTP  2078
NW_004765908_Musca    YKNGPPQEGDEG----EAAGGEDGAEGGEGEGGSGGGGGGGDDGGSATGATAAAAGATSP  2044
                      :*:      *. .     :*...:** :** * *..*..****    . ..*: : ..*.*:*
```

```
NC_035109_aegypt        GSGGGGSQANLGIVVEHNLSPKESPDGNNDPQGRQTAVLVESDGFVTKNGHRVVIHSRSP  2138
NW_004765908_Musca      SDPDAGEA--DGASVGGPLSPGCV---SGGSNGRQTAVLVESDGFVTKNGHKVVIHSRSP  2099
                        .. ..*.     *  *   ***      ... :*******************:********

NC_035109_aegypt        SITSRSADV     2147
NW_004765908_Musca      S--------     2100
                        *
```

Figure L. Sequence alignment of the Para protein of *Aedes aegypti* and *Musca domestica*. Kdr mutation sites are red and the respective location is given in boxes above and below the alignment.

City	Pesticides - Product Name	Pesticides - Active Ingredient
Las Cruces	not provided	Etofenprox
		Deltamethrin
		Chlorpyrifos
		Bifenthrin
		Lambda Cyhalothrin
T or C	Prentox Aqua Perm-X UL 30-30	Permethrin and Piperonyl Butoxide
	Altosid XR Briquets	(S)-Methoprene
	Aqualuer 20-20	Permethrin and Piperonyl Butoxide Technical
Roswell	no info	no info
Lordsburg	none	n/a
Alamogordo	Vectobac	*Bacillus thuringiensis* subsp. *israelensis*
Clovis	Delta Guard	Deltamethrin
	Atrapa	Malathion
Sunland Park	Altosid products (2015, 2016, 2017)	(S)-Methoprene
	Fyfanon (2016, 2017)	Malathion
	Kontrol 4x4 (2015)	Permethrin and Piperonyl Butoxide
	Vectolex CG products (2013, 2014)	*Bacillus sphaericus*
	Evoluer 30-30 (2013, 2014)	Permethrin and Piperonyl Butoxide
	Sustain MBG Pellets (2013)	*Bacillus thuringiensis* subsp. *israelensis*
Portales	no info	no info
Carlsbad	Mosquito Master	Chlorpyrifos and Permethrin
	Delta Guard	Deltamethrin
	Altosid	(S)-Methoprene
	Fourstar BTI	*Bacillus thuringiensis* subsp. *Israelensis*
	Natular™ products	Spinosad (soil bacterium)
	Permex	Permethrin
	Pursuit	Permethrin and Piperonyl Butoxide
	PyroFos 1.5 ULV (discontinued by the manufacturer)	Chlorpyrifos
Lovington	Mosquito Master	Chlorpyrifos and Permethrin
	BTI Briquits	*Bacillus thuringiensis* subsp. *Israelensis*
Deming	not provided	Etofenprox
		Deltamethrin
		Chlorpyrifos
		Bifenthrin
		Lambda Cyhalothrin
Socorro	not provided	Permethrin

Table A. Pesticide-use in selected cities across New Mexico.

City	# sites contributing to adults	# adults added to cage
Alamogordo	9	28
Carlsbad	8	144
Deming	6	28
Las Cruces	9	60
Lovington	5	20
Roswell	8	22
Sunland Park	12	72

Table B. Number of founding individuals for New Mexico *Aedes aegypti* strains used in this study. Traps were set for 24 hours at the respective sites.
